# Method of relieving alcohol dysphoria in the structure of hypertoxic alcohol abuse state with compulsive craving manifestations

**DOI:** 10.1192/j.eurpsy.2021.1499

**Published:** 2021-08-13

**Authors:** I. Sosin, G. Mysko, O. Sergienko, O. Honcharova, Y. Babenko, O. Minko

**Affiliations:** 1 Narcology Department, Kharkiv, Kharkiv Medical Academy of Postgraduate Education, Kharkiv, Ukraine; 2 Narcology Department, Kharkiv Medical Academy of Postgraduate Education, Kharkiv, Ukraine; 3 Department Of Urgent Psychiatry And Narcology, INPN NAMSU, Kharkiv, Ukraine

**Keywords:** Alcohol, Addiction, Dysphoria, Treatment

## Abstract

**Introduction:**

Alcohol dysphoria is a pathognomonic, severe, and therapeutically resistant syndrome considerable for alcohol and drug-addicted patients. The term “dysphoria” (from Greek δυσφορέω to suffer, torment, annoy) means an abnormally low type of mood, characterized by anger, gloom, irritability, feelings of hostility to others. In addictology, it is often identified in the withdrawal syndrome structure.

**Objectives:**

To develop innovative improvement in treatment for alcohol dysphoria.

**Methods:**

Valid clinical diagnostic, laboratory, biochemical, electrophysiological, psychological (scaling, testing), statistical methods identifying alcohol dependence complicated by dysphoria.

**Results:**

The proposed method involves a complex of anti-affective, anti-abstinence, anti-craving pharmacological agents and drug-free methods, and differs from those conventional, along with psychotherapeutic potentiation, by additional targeted pharmacological triad (peroral Carbamazepine 200 mg twice a day: in the morning and in the evening; intramuscular Halopril (Haloperidol) 1 ml (5 mg) daily; oral Sonapax 1 tablet (25 mg) three times a day for 3-5 day treatment) used for a new purpose. 17 patients experienced this method. Efficacy: alcohol dysphoria acute manifestations were relieved by our method within 3-5 days that 37.8% exceeds conventional treatment. In 15 minutes, patients decreased irritability, motor restlessness, stress, cravings for alcohol. In 30 minutes, the patients fell asleep. Sleep lasted 3.5 hours on average. Subsequently, patients denied craving for alcohol, calmed down emotionally and psychomotorically, wished to be treated for alcoholism. No dysphoric relapses were observed.

**Conclusions:**

The proposed multimodality method alleviates alcohol-induced dysphoria, involving pharmacotherapeutic triad along with psychotherapeutic potentiation.

